# MHC class I/II deficient NOG mice are useful for analysis of human T/B cell responses for human tumor immunology research

**DOI:** 10.1186/2051-1426-1-S1-P39

**Published:** 2013-11-07

**Authors:** Tomonori Yaguchi, Asuka Kobayashi, Ikumi Katano, Yuyo Ka, Mamoru Ito, Yutaka Kawakami

**Affiliations:** 1 Division of Cellular Signaling, Institute for Advanced Medical Research, Keio University School of Medicine, Shinjyuku-ku, Japan; 2 Central Institute for Experimental Animals, Kawasaki, Japan

## 

Immunologically humanized mice are promising tools to analyze in vivo human anit-tumor immune responses. When human PBMCs are transferred into NOG (NOD/Shi-scid IL2rγnull) mice, severe GVHD developed in a couple of weeks hinders long term detailed analysis of human immune responses. In this study, we developed novel MHC class I and class II-deficient NOG mice, and characterized the reconstituted human immune systems in the mice after inoculation of human PBMC. NOG mice with no murine MHC class I and class II were generated by knockout of β2-microglobulin gene, a component of MHC class I molecule, and IAβ, the light chain of the class II IA (NOG-dKO; NOG-β2m, IAβ double KO mouse). Administration of human PBMC into NOG-dKO (huNOG-dKO) mice induced mild GVHD, but its severity was much less than that occurred in control NOG mice in spite of sufficient engraftment of human immune cells, when evaluated by decrease of body weight and survival as well as human T cell infiltration in various organs. Various types of human immune cells were detected in the peripheral blood of NOG-dKO until day14 (DC and naïve T cells), day21 (NKT cells), day35 (NK cells), or more than day100 (T cells and B cells). Immunization with an inactivated influenza vaccine resulted in the increase of serum influenza-specific human IgG Ab along with increases of influenza-specific Ab producing B cells in the spleens, indicating the induction of antigen specific B cells in the huNOG-dKO. Immunization with human monocyte-derived dendritic cells (DC) pulsed with HLA-A2 restricted CMV peptide (CMVpp65) induced CMVpp65-specific CTLs in spleens, bone marrows, and peripheral blood, indicating the induction of antigen specific T cells in the huNOG-dKO. When activated human peripheral blood T cells transduced with TCR specific for melanoma antigen MART-1, were transferred into NOG-dKO and NOG mice implanted with human melanoma cell lines, stronger tumor growth inhibition was observed in the NOG mice along with more increased transferred T cells due to xenogenic GVHD responses than the NOG-dKO mice, and the NOG mice died in 4-8 weeks. However, administration of human recombinant IL2 into the NOG-dKO mice resulted in enhanced anti-tumor effects without any sign of GVHD accompanied by prolonged survival of T cells containing higher MART-1/HLA-A2 tetramer positive cells, indicating more detailed analysis of anti-tumor immune responses is possible using NOG-dKO mice. Therefore, NOG-dKO mice are useful tools for more detailed analysis of both induction and effector phases of T cell and B cell responses for human tumor immunology research.

